# Inflammation as a Regulator of the Airway Surface Liquid pH in Cystic Fibrosis

**DOI:** 10.3390/cells12081104

**Published:** 2023-04-07

**Authors:** Tayyab Rehman, Michael J. Welsh

**Affiliations:** 1 Department of Internal Medicine, University of Michigan, Ann Arbor, MI 48109, USA; 2 Departments of Internal Medicine and Molecular Physiology and Biophysics, Pappajohn Biomedical Institute, Roy J. and Lucille A. Carver College of Medicine, University of Iowa, Iowa City, IA 52242, USA; 3 Howard Hughes Medical Institute, University of Iowa, Iowa City, IA 52242, USA

**Keywords:** airway surface liquid, cystic fibrosis, inflammation, host defense, pH, airway epithelium

## Abstract

The airway surface liquid (ASL) is a thin sheet of fluid that covers the luminal aspect of the airway epithelium. The ASL is a site of several first-line host defenses, and its composition is a key factor that determines respiratory fitness. Specifically, the acid–base balance of ASL has a major influence on the vital respiratory defense processes of mucociliary clearance and antimicrobial peptide activity against inhaled pathogens. In the inherited disorder cystic fibrosis (CF), loss of cystic fibrosis transmembrane conductance regulator (CFTR) anion channel function reduces HCO_3_^−^ secretion, lowers the pH of ASL (pH_ASL_), and impairs host defenses. These abnormalities initiate a pathologic process whose hallmarks are chronic infection, inflammation, mucus obstruction, and bronchiectasis. Inflammation is particularly relevant as it develops early in CF and persists despite highly effective CFTR modulator therapy. Recent studies show that inflammation may alter HCO_3_^−^ and H^+^ secretion across the airway epithelia and thus regulate pH_ASL_. Moreover, inflammation may enhance the restoration of CFTR channel function in CF epithelia exposed to clinically approved modulators. This review focuses on the complex relationships between acid–base secretion, airway inflammation, pH_ASL_ regulation, and therapeutic responses to CFTR modulators. These factors have important implications for defining optimal ways of tackling CF airway inflammation in the post-modulator era.

## 1. Introduction

The airway surface liquid (ASL) is a thin layer of fluid that covers the luminal aspect of the airway epithelium [1,2,3]. The ASL thus forms a point of contact with the environment and is a site of several first-line host defenses. Antimicrobial peptides within ASL disrupt microbial cell membrane integrity, and thereby kill inhaled pathogens [4]. Gel-forming mucins, secreted into ASL, engage inhaled particles and pathogens, and the coordinated beating of cilia removes them from the lungs (mucociliary clearance) [5]. Neutrophils and macrophages phagocytose microbes that settle within ASL or kill them by extruding a meshwork of chromatin fibers [6,7]. Reactive oxygen species, released into ASL, suppress bacterial growth [8,9], and secreted purinergic nucleotides regulate ASL volume [10,11]. Several ASL factors (e.g., lysozyme, LL-37) show antiviral activity [12], and extracellular proteases alter the infectivity of respiratory viruses [13,14]. ASL composition critically influences these processes, and thus determines respiratory fitness. For the purpose of this review, we focus on the acid–base balance of ASL as a key parameter that controls host defense properties. Other factors that have been hypothesized to influence ASL properties include increased activity of the epithelial Na^+^ channels [15] and DNA accumulation [16,17].

**Figure 1:**
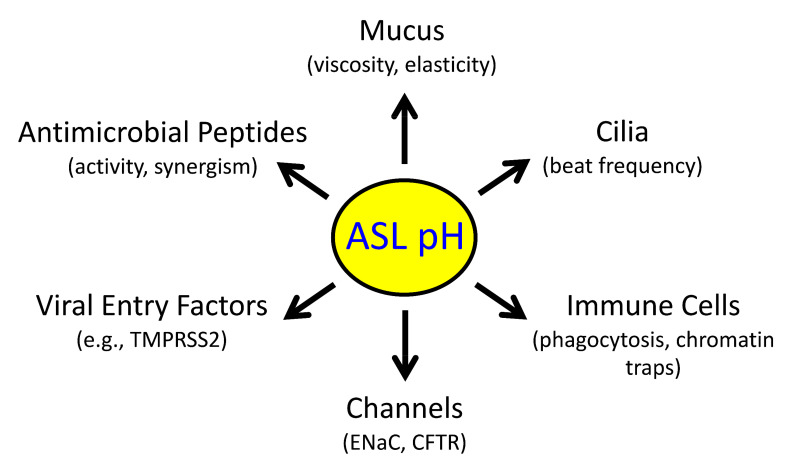
pH_ASL_ influences respiratory host defenses. The acid–base balance of ASL controls key first-line airway host defenses including secreted antimicrobial peptide activity and synergism against inhaled bacteria; mucus viscosity and elasticity; ciliary beat frequency (CBF)^#^; innate immune cell activities such as phagocytosis and extracellular killing of microbes through release of chromatin; activities of apical channels (e.g., acidic pH_ASL_ inhibits short palate lung and nasal epithelial clone 1-mediated inhibition of ENaC, promoting increased Na^+^ absorption [28]; extracellular HCO_3_^−^ concentration, sensed by soluble adenylyl cyclase, regulates CFTR expression [33,34]); and entry of respiratory viruses into airway epithelial cells (e.g., pH-dependent entry of SARS-CoV-2 in TMPRSS2-expressing cells [35])^##^. ASL = airway surface liquid, ENaC = epithelial Na^+^ channel, CFTR = cystic fibrosis transmembrane conductance regulator, TMPRSS2 = transmembrane serine protease 2. ^#^The mechanism by which pH_ASL_ alters CBF is not clear. In one study, CBF in bronchial cells increased as extracellular pH increased from 6 to 7.5 [26]. However, pH outside this range reduced CBF. Interestingly, the effect was less prominent in small airway ciliated cells. ^##^Most studies of airway physiology use proximal (large) airway cells. As CF airway disease involves distal (small) airways, regional differences in pH_ASL_ regulation and host defense mechanisms require further attention.

The abnormal acidification of ASL initiates a pathologic process in the airways, and for some lung disorders may provide a therapeutic target [18]. A preeminent example is cystic fibrosis (CF), an inherited disorder caused by mutations in the *cystic fibrosis transmembrane conductance regulator* (*CFTR*) gene [19,20]. The protein encoded by this gene forms an anion channel that conducts Cl^−^ and HCO_3_^−^ across the apical membrane of several epithelia, including airway epithelia [21,22]. *CFTR* mutations eliminate or markedly reduce anion flow, thereby decreasing HCO_3_^−^ secretion and lowering the pH of ASL (pH_ASL_). The abnormally acidic ASL impairs, at least in part, an array of first-line host defenses (Figure 1) [23,24,25,26,27,28,29]. With time, CF airways develop bacterial and viral infection and inflammation. Inflammation in turn can alter acid–base secretion and pH_ASL_. In recent years, the development of CFTR modulators has dramatically improved respiratory outcomes in people with CF [30,31,32]. However, airway inflammation persists in most people taking modulators. Importantly, inflammation can modify the response of CF airway epithelia to CFTR modulators. The complex interplay of these factors has important implications for how we approach CF airway inflammation in the post-modulator era. This review begins by outlining the cellular and molecular mechanisms that control pH_ASL_. We focus on the relationships between inflammation, pH_ASL_ regulation, and responses to CFTR modulators. We conclude by exploring the relevance of these findings for targeting CF airway inflammation.

## 2. H^+^ and HCO_3_^−^ Transporters Control pH_ASL_

**Figure 2:**
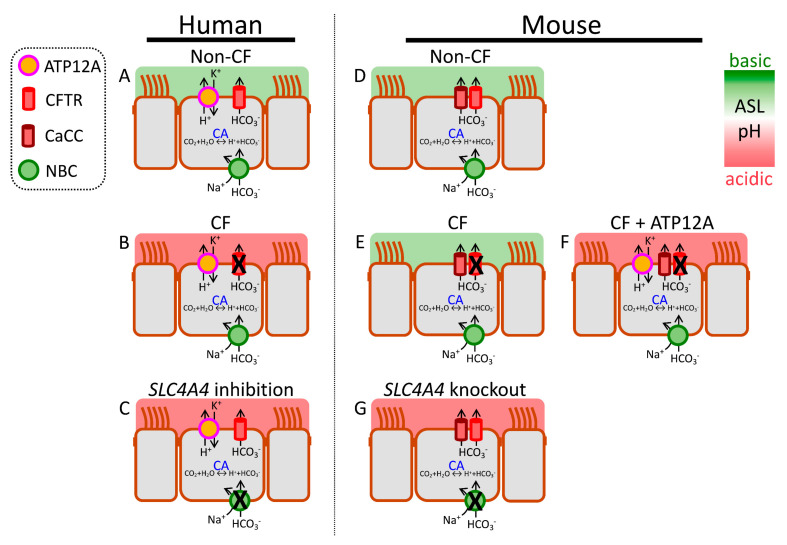
Acid–base transporters that control pH_ASL_ differ between human and mouse airways. Models show key transport mechanisms that determine pH_ASL_ in human and mouse airway epithelia. Left panel (human): (**A**) a model of non-CF airway epithelium with ASL overlying the apical membrane; (**B**) loss of CFTR-mediated HCO_3_^−^ secretion resulting in a lower pH_ASL_; (**C**) inhibition of basolateral NBC diminishes HCO_3_^−^ secretion and lowers pH_ASL_ despite intact apical CFTR channels. Right panel (mouse): (**D**) a model of non-CF (wild type) mouse airway epithelium. Note the absence of ATP12A and the expression of non-CFTR (CaCC) HCO_3_^−^ channels; (**E**,**F**) in contrast to humans, loss of CFTR fails to lower pH_ASL_ in CF mice, providing one explanation for lack of spontaneous airway disease. However, exogenous ATP12A expression increases H^+^ secretion and lowers CF mouse pH_ASL_; (**G**) *SLC4A4^−/−^* mice phenocopy human CF. For simplicity, only the chief acid–base transport mechanisms controlling pH_ASL_ are shown. We do not show Na^+^/H^+^ exchangers, Cl^−^/HCO_3_^−^ exchangers, Na^+^ and K^+^ channels, or Na^+^/K^+^-ATPase, which may also influence the movement of acid–base equivalents into or out of ASL. See legend and text for details. CA = carbonic anhydrase.

The balance of acid (H^+^) and base (HCO_3_^−^) secretion across the apical membrane determines pH_ASL_ [36,37]. In the proximal (cartilaginous) airways, the non-gastric H^+^/K^+^-ATPase (ATP12A) is the main route for H^+^ secretion, and CFTR is the main route for HCO_3_^−^ secretion. Non-CFTR mechanisms such as calcium-activated Cl^−^ channels (CaCC) and solute carrier family 26 (SLC26) transporters are also capable of secreting HCO_3_^−^, but their overall contribution is small compared to CFTR (Figure 2) [36,38,39,40]. In contrast, in the distal (small) airways, ATP12A is absent, and the vacuolar H^+^-ATPase (V-ATPase) substitutes as the main H^+^-secreting mechanism [41]. Both CFTR and CaCC mediate HCO_3_^−^ secretion across small airway epithelia [42].

Two distinct mechanisms provide HCO_3_^−^ to apical HCO_3_^−^ channels and transporters. First is the Na^+^-HCO_3_^−^ cotransport (NBC) activity located at the basolateral membrane [43,44,45]. NBC activity is mediated by solute carrier 4 (SLC4) family transporters, several of which are expressed in airway epithelia. These transporters employ the favorable Na^+^ gradient to cotransport HCO_3_^−^ into the cytosol with a stoichiometry varying between 1:1 to 1:3, depending on the NBC isoform. However, molecular mechanisms regulating basolateral NBC activity in human airway epithelia remain poorly understood.

Another source of secreted HCO_3_^−^ is the CO_2_ hydration reaction [21,46]. The rate-limiting step in this reaction, i.e., the conversion of reactants H_2_O and CO_2_ to carbonic acid, is catalyzed by carbonic anhydrase (CA) enzymes. Once generated, carbonic acid readily dissociates into HCO_3_^−^ and H^+^. Several CA isoforms, both membrane-associated and cytoplasmic, are expressed in airway epithelia [40,47]. However, their contribution to overall HCO_3_^−^ secretion is small relative to that of NBCs.

Figure 2 shows a simple model of channels, transporters, and enzymes that control pH_ASL_ in airway epithelia. For clarity, we do not show the basolateral Na^+^/H^+^ exchanger (NHE1) or Cl^−^/HCO_3_^−^ exchanger (AE2), which play important roles in intracellular pH regulation. Nor do we show the Na^+^/K^+^-ATPase, and Na^+^ and K^+^ channels that influence HCO_3_^−^ flow indirectly by establishing and altering electrochemical gradients. Readers interested in these transport mechanisms are directed to excellent published reviews [36,37,43,48,49,50].

### 2.1. Reduced pH_ASL_ Disrupts Host Defenses

The normal pH_ASL_ is mildly acidic (e.g., 6.9–7.1 in human lower airways) relative to the interstitial fluid [7.4], though it varies considerably between individuals as well as between studies [1,36,37]. Recent reviews provide excellent tables summarizing pH_ASL_ variability between studies, including wild type versus CF [36,37]. Absolute pH_ASL_ measurements are influenced by technique, model system and airway region. It is also interesting to point out that pH is measured on log scale and a 3/10 increase in pH reflects a doubling of [H^+^].

Notwithstanding challenges involved in comparing results between studies, recent reports have identified several genetic changes in humans and/or animal models that alter pH_ASL_ (Figure 2). These studies provide critical insights into acid–base transport mechanisms that control pH_ASL_ and thus airway host defenses.

In humans with CF, the loss of CFTR-mediated HCO_3_^−^ secretion leaves H^+^ secretion unbalanced, and hence lowers pH_ASL_ [51]. An abnormally acidic pH_ASL_ impairs mucociliary clearance, antimicrobial peptide activity against inhaled pathogens, and phagocytic cell function [23,24,27]. Raising pH_ASL_ at least partially rescues these impairments and identifies epithelial acid–base transporters as potential therapeutic targets.

The involvement of pH_ASL_ in airway host defense has also been tested by disrupting acid–base transporters other than CFTR. For instance, inhibiting basolateral NBC activity in human airway epithelia with normal CFTR channels lowers pH_ASL_ [52]. Interestingly, *CFTR* disruption in mice fails to produce spontaneous airway disease [53,54]. This is partly due to increased expression of non-CFTR HCO_3_^−^ transporters and lack of expression of the non-gastric H^+^-pump (ATP12A) in murine airways (see below) [55]. As a result, pH_ASL_ in CF mice is the same as in non-CF mice. However, inhibiting basolateral NBC activity in freshly excised mouse tracheae reduces HCO_3_^−^ secretion [52]. Moreover, mice lacking the main NBC isoform (*SLC4A4^−/−^*) show an airway phenotype marked by thick, adherent mucus and reduced mucociliary clearance, changes reminiscent of human CF airway disease. 

Mutations in *carbonic anhydrase isoform 12* (*CA12*) also phenocopy the loss of CFTR channel activity in human airways [56]. People with *CA12* mutations show chronic coughs, airway colonization, bronchiectasis, and elevated sweat [Cl^−^]. Interestingly, CA12 localizes at the apical membrane of airway epithelia. This expression pattern is physiologically relevant because proximal airway CO_2_ concentration fluctuates during tidal breathing [57]. Thus, pH_ASL_ rises during inhalation as airway lumen fills up with inhaled air (low CO_2_ concentration) but falls during exhalation as alveolar gases (higher CO_2_ concentration) enter the airways. Tidal pH_ASL_ oscillations enhance the epithelial host defense against bacteria. In CF, the amplitude of pH_ASL_ oscillations is reduced, which suggests a potential mechanism linking CFTR loss with impaired host defense.

In addition to HCO_3_^−^ secretion, H^+^ secretion also controls pH_ASL_. As noted above, mouse airways lack ATP12A expression and pH_ASL_ in CF mice is not reduced [55]. However, exogenous expression of ATP12A in CF mice increases H^+^ secretion, lowers pH_ASL_, and impairs host defenses.

ASL buffers resist changes in pH_ASL_ when H^+^ ions are added or removed. The main ASL buffer is HCO_3_^−^ [58]. Accordingly, in Calu-3 epithelia, forskolin stimulation increases and CFTR knockdown decreases buffering capacity of apically secreted fluid. Mucins are negatively charged molecules that can also bind H^+^ and thus contribute to ASL buffering [59,60]. This effect is dependent on mucin concentration. Mucus accumulation lowers the amplitude of ventilatory pH_ASL_ oscillations [57], and dampened oscillations reduce antibacterial host defense, thus providing a potential mechanism by which mucus accumulation may increase susceptibility to respiratory infections.

Together, the studies highlighted above point to the vital role of HCO_3_^−^ and H^+^ transport mechanisms in controlling pH_ASL_. Abnormally acidic pH_ASL_, due to either reduced HCO_3_^−^ secretion or unbalanced/increased H^+^ secretion, impairs critical first-line airway host defenses and predisposes to processes such as inflammation and chronic bacterial colonization. In the next section, we review studies that investigate how pH_ASL_ in CF might change with the development of inflammation and progression of airway disease.

### 2.2. pH_ASL_ Changes with CF Airway Disease Progression

In vitro studies in human airway epithelia show that pH_ASL_ is abnormally acidic in CF [51,55,61], but in vivo studies reveal mixed results. Some studies show a lower pH_ASL_ in CF [62,63], whereas others report no difference between CF and non-CF individuals [64,65]. This discrepancy is intriguing, given that CFTR-mediated HCO_3_^−^ secretion is decreased in both in vitro and in vivo studies.

Most babies with CF develop airway inflammation over the first year of life [66,67,68,69,70]; in some cases, they also develop respiratory infections. Inflammation may induce ASL alkalinization through CFTR-independent mechanisms, and thus conceal the loss of CFTR-mediated HCO_3_^−^ secretion. Studying initial stages of human CF airway disease might help separate effects due to CFTR loss from those due to inflammation. As inflammation develops early in CF, this approach requires studying ASL from babies soon after birth. In one small pilot study, pH_ASL_ in newborn CF babies (<four weeks of age) was lower compared with non-CF babies [71]. However, when the investigators studied the same cohort at three months of age, pH_ASL_ in CF babies had increased and did not differ from pH_ASL_ in non-CF babies. These findings are intriguing but require validation in a larger, independent cohort.

Several observations from the CF pig model might be relevant here: newborn CF piglets lack airway inflammation and infection and have an abnormally acidic pH_ASL_ [23,72]. In contrast, three-week-old CF piglets have airway inflammation, and their pH_ASL_ is higher than at birth and not different from the non-CF pH_ASL_. Based on these observations, it may be predicted that CF-like inflammation in non-CF airways will further increase pH_ASL_, and that inflamed non-CF airways will have a higher pH_ASL_ than inflamed CF airways. However, this prediction remains untested, partly because experimentally reproducing CF-like inflammation in vivo remains a challenge.

Respiratory viral infections also develop early in CF and are often encountered during infancy [73]. CF epithelia show multiple defects in antiviral immunity including reduced ASL-mediated neutralization of respiratory viruses [12], impaired interferon signaling [74], and lower levels of nitric oxide and hypothiocyanite [9,75,76]. However, whether pH_ASL_ influences susceptibility to respiratory viruses or antiviral host defense is not clear. Conversely, whether viral infection or viral-induced inflammation change pH_ASL_ also remains unknown. 

CF airway inflammation is known to alter levels of airway antimicrobial peptides including defensins and LL-37 [77]. In addition to killing bacteria, antimicrobial peptides also modulate inflammation. For example, β-defensins interact with chemokine receptor 6 expressed on lymphocytes and dendritic cells and recruit these cells to sites of infection [78]. LL-37 directly binds LPS extracted from *Pseudomonas aeruginosa* and thus reduces IL-8 production from monocytes [79]. It is interesting to consider whether, similar to its effect on bacterial killing, pH_ASL_ also influences the immunomodulatory effects of antimicrobial peptides. Future studies in this area may reveal additional mechanisms linking reduced HCO_3_^−^ secretion and abnormal pH_ASL_ regulation in CF with impaired host defenses.

### 2.3. Inflammatory Cytokines Regulate pH_ASL_

**Figure 3:**
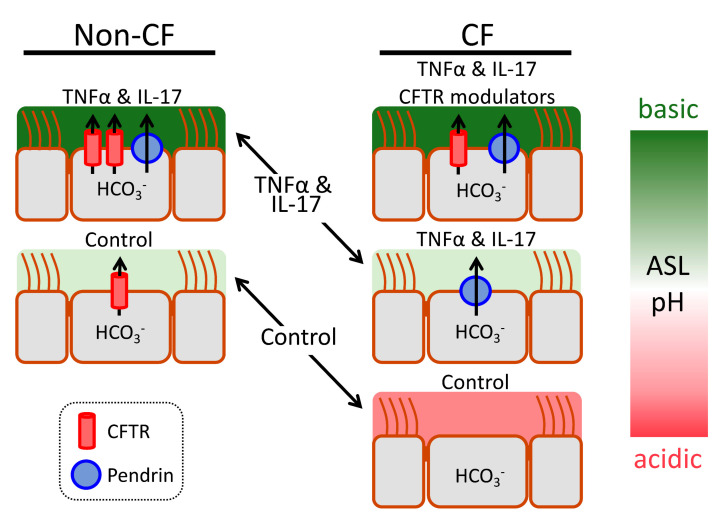
Inflammatory cytokines (IL-17/TNFα) regulate pH_ASL_ in human CF and non-CF airway epithelia. Control CF epithelia lack CFTR-mediated HCO_3_^−^ secretion and have a lower pH_ASL_ than control non-CF epithelia. IL-17/TNFα upregulate pendrin, an apical Cl^−^/HCO_3_^−^ exchanger, and thereby increase CF pH_ASL_. Non-CF epithelia exposed to IL-17/TNFα have a higher pH_ASL_ compared to similarly treated CF epithelia. Interestingly, restoring CFTR channel function in IL-17/TNFα-treated CF epithelia further increases pH_ASL_. Thus, maximal ASL alkalinization response involves two apical HCO_3_^−^ transporters, CFTR and pendrin. Modified from Rehman et al. [39] and reproduced with permission.

In the absence of rigorous in vivo assessments, the effects of inflammation on pH_ASL_ may be investigated in vitro by applying CF-relevant inflammatory stimuli to primary cultures of differentiated human airway epithelia. The cytokine interleukin-17 (IL-17) is an evolutionarily conserved molecule that drives neutrophilic airway inflammation [80,81]. In targeting the airway epithelium, IL-17 acts synergistically with other cytokines such as tumor necrosis factor-α (TNFα), IL-1β, etc. [82]. These proinflammatory molecules are increased in established CF airway disease [83,84]. Some studies have also evaluated the effects of IL-4 and IL-13, which are relevant to asthma and allergic bronchopulmonary aspergillosis, conditions that often affect CF individuals [47,85,86,87]. These in vitro studies have provided key insights into the cellular and molecular mechanisms by which inflammation might regulate pH_ASL_ (Figure 3). In the following section, we review salient findings from these studies.

### 2.4. H^+^ Secretion

Inflammation may alter acid secretion into ASL. One study reported increased expression of ATP12A in CF airways with established disease [88]. Exposure of airway epithelia to IL-4 or IL-13 also increased ATP12A expression, and thus H^+^ secretion [47,86]. TNFα exposure had a similar effect, but IL-17 alone or combined IL-17/TNFα did not change H^+^ secretion [40]. Importantly, inhibiting ATP12A with apical ouabain decreases H^+^ secretion, lowers ASL viscosity, and increases bacterial killing [51,55,86]. Efforts to identify safer agents that reduce ATP12A activity are underway. 

Loss of CFTR function also affects distal (small) airways. Small airway epithelia lack ATP12A, but instead use V-ATPase to secrete H^+^. Effects of inflammation and infection on small airway H^+^ secretion remain relatively unexplored. *P. aeruginosa* infection may reduce V-ATPase-mediated H^+^ secretion [89,90], or acidify ASL via apically expressed monocarboxylate transporter 2, a H^+^-lactate cotransporter [91]. Interestingly, Li et al. showed that pH_ASL_ regulates membrane localization of V-ATPase in porcine small airway epithelia [41]. At neutral pH_ASL_ (7.4), the V-ATPase subunit ATP6V0D2 localizes in the apical membrane of small airway secretory cells. However, at a lower extracellular pH of 6.8, ATP6V0D2 translocates into cytosol. V-ATPase may thus alter its apical membrane location and activity to prevent large changes in pH_ASL_. Additional studies are needed to understand H^+^ secretion and pH_ASL_ regulation in small airway epithelia under both basal and inflamed conditions.

### 2.5. HCO_3_^−^ Secretion

#### 2.5.1. CFTR-Mediated HCO_3_^−^ Secretion

Several inflammatory cytokines have been shown to alkalinize pH_ASL_. IL-17/TNFα, IL-4, IL-13, and IL-1β increase CFTR expression and activity, and increased CFTR-mediated anion secretion improves respiratory host defenses [40,47,92,93]. In CF epithelia, this component of host response is missing due to mutated, non-functional CFTR proteins. In contrast to above-mentioned cytokines, TGF-β reduces CFTR expression and transport activities in airway epithelia [94,95]. In vivo effects of inflammation are likely to be complex given that several cytokines elevated in CF airways target airway epithelium, alter HCO_3_^−^ transport, and thus modify pH_ASL_.

#### 2.5.2. Non-CFTR-Mediated HCO_3_^−^ Secretion

An array of cytokines (IL-17/TNFα, IL-4, and IL-13) induce non-CFTR HCO_3_^−^ secretion across airway epithelia [39,47,85,92,96]. This is achieved through pendrin, an apical Cl^−^/HCO_3_^−^ exchanger, encoded by the gene *SLC26A4*. Several aspects of this transport process are noteworthy. First, pendrin is minimally expressed in airway epithelia under basal conditions but is strongly upregulated by inflammatory cytokines. Second, pendrin is an electroneutral exchanger that does not mediate net anion secretion or absorption or change membrane potential. Third, in the absence of functional CFTR channels, pendrin alone can drive ASL alkalinization, though greater alkalinization is achieved with pendrin plus CFTR [39,40]. Fourth, some reports indicate structural or functional interactions between CFTR and pendrin, resulting in the increased activity of both [85,97]. Although these transporters are coexpressed in secretory cells, and studies in heterologous expression systems are supportive, more evidence is needed to establish their significance. Potentiating pendrin-mediated HCO_3_^−^ secretion is a promising strategy for alkalinizing ASL and might be particularly relevant to airway inflammatory disorders.

#### 2.5.3. Paracellular HCO_3_^−^ Shunt

In addition to secretion by airway epithelial cells, HCO_3_^−^ can also move between the cells. Very few studies have explored the contribution of the paracellular pathway to pH_ASL_, though it is often mentioned in the context of inflammation. A recent report showed that the paracellular pathway is as permeable to HCO_3_^−^ as it is to Cl^−^ [98]. Under basal conditions, pH_ASL_ (6.6) is lower than the pH of the interstitial fluid (7.4) and the paracellular HCO_3_^−^ flux is towards the lumen; however, the paracellular HCO_3_^−^ flux decreases or even reverses as pH_ASL_ approaches or rises higher than the pH of the interstitial fluid. The paracellular pathway thus acts as a HCO_3_^−^ shunt that may oppose increased cellular HCO_3_^−^ secretion in inflamed airway epithelia [99]. Whether paracellular HCO_3_^−^ permeability can be modulated to support ASL alkalinization is an interesting question and requires further investigation. 

### 2.6. Other Regulatory Mechanisms

**Figure 4:**
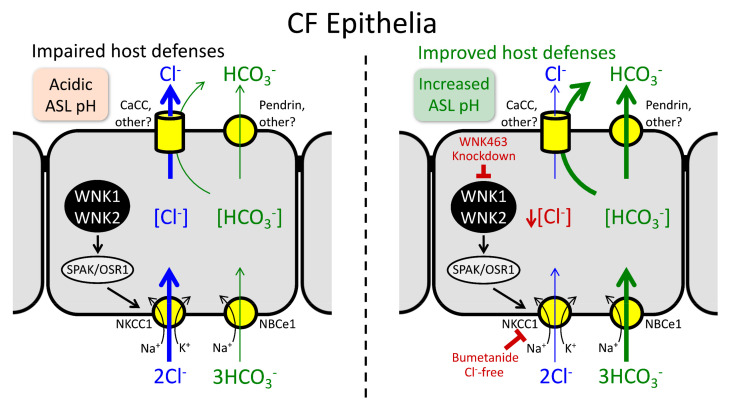
WNK kinases regulate HCO_3_^−^ versus Cl^−^ secretion across human airway epithelia. Model shows the with-no-lysine [K] (WNK) kinase signaling pathway in human CF airway epithelia lacking functional CFTR. Left panel: WNK1 and WNK2 signal via intermediate Ste20/SPS1-related proline-alanine-rich protein kinase/oxidative stress responsive 1 kinase (SPAK/OSR1) to regulate basolateral Na^+^-K^+^-2Cl^−^ cotransporter (NKCC1) activity. Right panel: reducing WNK activity, inhibiting NKCC1, or removing Cl^−^ from basolateral solution lower the intracellular [Cl^−^]. At the same time, these interventions also increase HCO_3_^−^ secretion and alkalinize ASL. Higher pH_ASL_ improves epithelial host defenses which are otherwise impaired in CF. The mechanism by which WNK kinases and intracellular [Cl^−^] regulate apical and/or basolateral HCO_3_^−^ transporters (CaCC, pendrin, NBC) remain unknown. Modified from Rehman et al. [101] and reproduced with permission. Copyright © 2022 American Thoracic Society. All rights reserved. The American Journal of Respiratory Cell and Molecular Biology is an official journal of the American Thoracic Society.

Inflammatory cytokines regulate diverse cellular mechanisms involved in HCO_3_^−^ secretion. In addition to changes in apical HCO_3_^−^ transporters, cytokines such as IL-17/TNFα, IL-13, or IL-4 also increase transcripts of several carbonic anhydrase and NBC isoforms [39,47]. Additional cytoplasmic mechanisms that regulate epithelial HCO_3_^−^ secretion also change in the presence of cytokines. The WNK (with-no-lysine [K]) kinases are master-regulators of pancreatic HCO_3_^−^ secretion [100]. As reported recently, these kinases also control HCO_3_^−^ secretion across CF and non-CF airway epithelia [101]. Secretory cells, key HCO_3_^−^ secreting cells in airway epithelia, express two WNK isoforms, WNK1 and WNK2. Reducing WNK kinase activity increases HCO_3_^−^ secretion, raises pH_ASL_, and enhances CF epithelial host defenses. At a mechanistic level, WNK kinases regulate intracellular [Cl^−^] through their control of the basolateral Na^+^-K^+^-2Cl^−^ cotransporter (NKCC1) (Figure 4). Inhibiting WNK lowers intracellular [Cl^−^], which in turn may act as a signaling ion to stimulate HCO_3_^−^ transport [102,103]. It is of note that combined IL-17/TNFα reduce WNK2 expression and raise pH_ASL_ and inhibiting residual WNK kinase activity further alkalinizes ASL. Future investigations may reveal additional mechanisms that regulate HCO_3_^−^ and H^+^ secretion in inflamed airway epithelia.

## 3. Airway Inflammation and CFTR Modulators Influence Each Other

The natural history of CF airway disease has changed markedly with the widespread use of highly effective CFTR modulator therapy (HEMT) [104,105,106,107,108]. CFTR modulators are small-molecule drugs used to restore anion channel activity to mutated CFTR proteins. These agents include potentiators which increase the open-state probability of CFTR channels at the apical membrane, and correctors which increase the processing of misfolded CFTR proteins. A remarkably striking proof of the real-world effectiveness of these agents is the precipitous decline in new lung transplants for CF individuals coinciding with the availability of HEMT for > 90% of people with CF [109,110].

Inflammation is highly prevalent in CF individuals taking HEMT, which makes the relationship between airway inflammation and CFTR modulators critically important. The nature of this relationship is two-fold. On the one hand, starting HEMT results in either no change or a decrease in airway inflammatory markers [111,112,113]. On the other, inflammatory mediators that are elevated in CF airways enhance the restoration of CFTR channel function in response to CFTR modulators [39,114,115]. Interestingly, CFTR modulators further alkalinize pH_ASL_ in IL-17/TNFα-treated CF epithelia, but not in control CF epithelia [39]. Moreover, baseline sputum inflammatory markers (IL-8, IL-1β, neutrophil elastase) positively correlate with CFTR modulator-induced lung function improvements [39]. These effects are consistent with robust clinical improvements observed in most CF individuals after starting HEMT [109].

It is interesting to speculate as to the long-term effects of HEMT on pH_ASL_. As noted above, pendrin is minimally expressed under basal conditions but markedly upregulated by inflammation. In inflamed CF airways, HEMT may further influence pH_ASL_ through at least two mechanisms: (a) by adding functional CFTR channels; and (b) by changing the level of inflammation and perhaps pendrin expression. Over time, how these factors might influence net HCO_3_^−^ secretion and the relative contribution of CFTR and pendrin is not clear and is an interesting question for future studies.

## 4. Optimal Anti-Inflammatory Strategy in CF Is Unclear

Airway inflammation and bacterial colonization persist even after prolonged use of HEMT [116,117]. Considerable evidence suggests that inflammation may contribute to CF airway pathology [118]. However, the reports reviewed above indicate that inflammation may also have at least two beneficial effects: (1) it increases pH_ASL_ that may partially rescue host defense, and (2) it increases the response to CFTR modulators. Some studies suggest that cellular pathways activated by inflammation intersect with those involved in CFTR biogenesis [119]. It follows that the use of non-specific anti-inflammatory agents may limit ASL alkalinization and the restoration of CFTR channel activity with HEMT. 

In the past, anti-inflammatory therapies in CF have produced mixed results. High-dose ibuprofen is currently the only strategy recommended by treatment guidelines, but it is not widely pursued in real-world settings due to issues with compliance and side-effects [120,121,122,123]. Other agents such as corticosteroids, azithromycin, etc., have not proved consistently effective. In airway epithelia, glucocorticoids reduce anion secretion [124] and *CFTR* mRNA expression [125]. Systemic steroids are well known to induce hyperglycemia, which may increase ASL glucose concentration, lower pH_ASL_, and promote bacterial survival [91]. Considering these complex, inconsistent, and potentially harmful effects, the optimal strategy to target residual airway inflammation in people taking HEMT remains unclear. For future anti-inflammatory agents entering preclinical or clinical evaluation, reducing inflammation-mediated tissue damage without compromising HCO_3_^−^ secretion, *CFTR* expression, and responses to CFTR modulators are all desirable features.

## 5. Conclusions

A well-orchestrated host response to inhaled particles and pathogens is essential for respiratory fitness. In CF, impaired HCO_3_^−^ secretion and an abnormally acidic pH_ASL_ disrupt innate mucosal host defenses and initiate airway pathology. Persistent environmental challenges lead to chronic inflammation, a complex process with both protective and pathogenic effects. These effects are particularly important in the setting of bacterial or fungal colonization. Inflammation and infection persist after long-term HEMT and thus remain relevant concerns in the post-modulator era. However, the optimum anti-inflammatory approach for people taking HEMT remains unclear. One goal for future research might be to reveal targets and strategies that limit tissue damage but preserve HCO_3_^−^ secretion and therapeutic responses to CFTR modulators. This goal may be pursued using a combination of in vitro and in vivo approaches. In vitro models may help achieve a better understanding of signaling pathways involved in acid–base secretion, CFTR biogenesis, and their relationship to inflammation. In vivo CF models that develop airway inflammation and have mutations amenable to CFTR modulators may prove useful in evaluating the safety and efficacy of novel anti-inflammatory agents.

## Data Availability

Not applicable.
